# An analysis of *P* *seudomonas* genomic diversity in take‐all infected wheat fields reveals the lasting impact of wheat cultivars on the soil microbiota

**DOI:** 10.1111/1462-2920.13038

**Published:** 2015-10-06

**Authors:** T. H. Mauchline, D. Chedom‐Fotso, G. Chandra, T. Samuels, N. Greenaway, A. Backhaus, V. McMillan, G. Canning, S. J. Powers, K. E. Hammond‐Kosack, P. R. Hirsch, I. M. Clark, Z. Mehrabi, J. Roworth, J. Burnell, J. G. Malone

**Affiliations:** ^1^ Department of AgroEcologyRothamsted ResearchHarpendenUK; ^2^ Department of Plant Biology and Crop ScienceRothamsted ResearchHarpendenUK; ^3^ Department of Computational and Systems BiologyRothamsted ResearchHarpendenUK; ^4^ The Wellcome Trust Centre for Human Genetics; ^5^ Oxford Long‐term Ecology and Resource Stewardship LabUniversity of OxfordOxfordUK; ^6^ Molecular Microbiology DepartmentJohn Innes CentreNorwichUK; ^7^ School of Biological SciencesUniversity of East AngliaNorwichUK; ^8^ School of Physics and AstronomyUniversity of EdinburghEdinburghUK

## Abstract

Manipulation of the soil microbiota associated with crop plants has huge promise for the control of crop pathogens. However, to fully realize this potential we need a better understanding of the relationship between the soil environment and the genes and phenotypes that enable microbes to colonize plants and contribute to biocontrol. A recent 2 years of investigation into the effect of wheat variety on second year crop yield in the context of take‐all fungal infection presented the opportunity to examine soil microbiomes under closely defined field conditions. Amplicon sequencing of second year soil samples showed that *P* *seudomonas* spp. were particularly affected by the wheat cultivar grown in year one. Consequently, 318 rhizosphere‐associated *P* *seudomonas fluorescens* strains were isolated and characterized across a variety of genetic and phenotypic traits. Again, the wheat variety grown in the first year of the study was shown to exert considerable selective pressure on both the extent and nature of *P* *seudomonas* genomic diversity. Furthermore, multiple significant correlations were identified within the phenotypic/genetic structure of the *Pseudomonas* population, and between individual genotypes and the external wheat field environment. The approach outlined here has considerable future potential for our understanding of plant–microbe interactions, and for the broader analysis of complex microbial communities.

## Introduction

Manipulation and supplementation of the soil microbiota has significant potential for the development of natural treatments to control diseases such as take‐all (*Gaeumannomyces graminis* var. *tritici*); an important fungal crop pathogen that affects around half of UK wheat crops and causes substantial yield losses, particularly in temperate climates (Hornby and Bateman, 1998; Bateman *et al*., 2004). The Gram‐negative, soil‐dwelling bacterium *Pseudomonas fluorescens* is an important biocontrol organism in numerous different plant–microbe systems (Naseby *et al*., 2001; Haas and Defago, 2005). The established role of *P. fluorescens* in take‐all suppressive soils (Mazzola and Cook, 1991; Cook *et al*., 1995; Bergsma‐Vlami *et al*., 2005; Yang *et al*., 2011; Kwak and Weller, 2013) makes this disease a highly attractive target for the development of *Pseudomonas* biocontrol agents. However, the effects of *Pseudomonas* biocontrol are currently somewhat inconsistent (Weller, 1988). A major reason for this is an incomplete understanding of the phenotypic characteristics relevant for biocontrol and colonization in different plant environments. The issue is further complicated by the extensive genetic diversity found within the *Pseudomonas* species group, with the core genome representing as little as 45% of an individual bacterial genome (Silby *et al*., 2009; Loper *et al*., 2012; Redondo‐Nieto *et al*., 2012; Seaton and Silby, 2014).

*Pseudomonas fluorescens* non‐specifically colonizes plant rhizospheres, exploiting root exudates as a nutrient and energy source. The colonization process is highly regulated, from initial migration into the root zone to the formation of a bacterial biofilm (Chin‐A‐Woeng *et al*., 1997). The initial stages of plant association rely on motility systems including flagella, type IV pili and biosurfactants, which facilitate swarming (Lugtenberg *et al*., 2001; Alsohim *et al*., 2014). Once at the root surface, exopolysaccharides including alginate and cellulose play important roles in rhizoplane attachment and biofilm formation (Chin‐A‐Woeng *et al*., 1997; Gal *et al*., 2003; Ma *et al*., 2006). Proteinaceous adhesion factors (Hinsa *et al*., 2003; Latasa *et al*., 2005; Borlee *et al*., 2010; Dueholm *et al*., 2010) and lipopolysaccharide biosynthesis (de Weert *et al*., 2006) also contribute to effective root colonization.

*Pseudomonas* spp. communicate with their host plants and compete with other members of the soil microbiome to successfully colonize the rhizosphere environment (Compant *et al*., 2010). They produce various secreted molecules to kill or suppress pathogenic fungi, bacteria and oomycetes, alongside insects and other plant predators (Haas and Defago, 2005; Loper *et al*., 2012). These molecules include siderophores, which are potent iron/metal scavengers that may inhibit pathogenic fungal growth by inducing metal ion limitation in the rhizosphere (Kloepper *et al*., 1980), cyclic lipopeptides to enable swarming motility (Alsohim *et al*., 2014) and solubilize pathogen cell membranes (de Bruijn *et al*., 2007), and phenazines – flavin coenzyme analogues that inhibit electron transport in plant pathogens and catalyse hydroxyl radical formation (Britigan *et al*., 1992; Ran *et al*., 2003). The *phz* locus has been linked to *Pseudomonas* ecological competence in take‐all infected wheat rhizospheres (Mazzola *et al*., 1992). Other antimicrobials synthesized by soil *Pseudomonas* spp. include phloroglucinols (Shanahan *et al*., 1992), the antifungal compounds pyoluteorin and pyrrolnitrin (Kirner *et al*., 1998; Nowak‐Thompson *et al*., 1999), and the volatile metalloenzyme inhibitor hydrogen cyanide (Blumer and Haas, 2000).

In addition to secondary metabolites, soil *Pseudomonas* spp. make various secreted toxins, pesticidal proteins and bacteriocins such as pyocin and LlpA (Parret and De Mot, 2002). Bacteriocins from commensal bacteria have been shown to contribute to biocontrol by killing related phytopathogenic species in model plant systems (Hert *et al*., 2009). Genes encoding insecticidal toxins, for example the *mcf*, *Tc* and *fit* operons, are also found in many *Pseudomonas* genomes (Loper *et al*., 2012; Pechy‐Tarr *et al*., 2013). *Pseudomonas* spp. deploy these extracellular molecules through various protein secretion pathways. Type II secretion systems are generally protein exporters and facilitate pathways as diverse as bacteriocin export and LapA adhesin secretion (Hinsa *et al*., 2003). Type III and Type VI complexes inject toxins and effector proteins into both eukaryotic and bacterial cells and are responsible for a range of cytotoxicity and virulence‐associated phenotypes (Hayes *et al*., 2010).

*Pseudomonas* spp. also produce various exoenzymes in order to adapt to the rhizosphere environment. These include proteinases, plant tissue‐degrading lyases, and chitinases, which contribute to biocontrol by hydrolysing fungal cell walls (Liao *et al*., 1994; Ayyadurai *et al*., 2007). Certain enzymes secreted by *Pseudomonas* spp. affect the behaviour of other organisms in the rhizosphere. For example, the protease AprA degrades monomeric flagellin and suppresses the plant immune response (Bardoel *et al*., 2011; Pel *et al*., 2014), whereas AHL Lactonases disrupt pathogen quorum sensing (Jafra *et al*., 2006). Finally, *Pseudomonas* spp. encode various plant–microbe communication pathways. Genes for the synthesis and catabolism of auxins (Loper *et al*., 2012), volatile plant‐growth‐promoting agents such as acetoin and 2‐3‐butanediol (Ryu *et al*., 2003) and the enzyme ACC deaminase, which suppresses ethylene production and protects the plant from environmental stress (Klee *et al*., 1991) have all been identified in soil *Pseudomonas* genomes.

Given the huge genetic variability within the *P. fluorescens* species group, examining the relationship between defined field environments and the abundance and distribution of different *Pseudomonas* genotypes has considerable promise for our understanding of how environmental changes affect bacterial genomes in the microbiota, with potential impacts both for the development of better crop management strategies and/or biocontrol treatments. A recent 2 year investigation into the effect of high and low take‐all inoculum building wheat varieties on crop yield in the second wheat (McMillan *et al*., 2011) presented the opportunity to examine the associations between *Pseudomonas* genes and phenotypes under defined environmental conditions, in the context of infection with an important crop pathogen. In this study, 318 *Pseudomonas* rhizosphere and endosphere isolates were collected from the second year wheat crop and subjected to extensive phenotypic and genetic analysis. From these, 19 representative genomes were sequenced, assembled and partially annotated to determine the presence or absence of loci associated with rhizosphere colonization and biocontrol. Phylogenetic analysis of the original 318 isolates, based on Enterobacterial Repetitive Intergenic Consensus (ERIC) polymerase chain reaction (PCR) profiles and housekeeping gene sequences showed substantial clustering of isolates based on the variety of wheat planted in year one, but not year two. The phenotypic and genomic data sets were then interrogated to identify correlations between phenotypes, genotypes, and the wheat varieties from which the strains were initially isolated. We observed very extensive phenotypic and genetic variation within the *Pseudomonas* population, with the diversity within a single field only slightly lower than that recorded for all *P. fluorescens* strains annotated to date. Furthermore, a number of significant correlations were identified between different phenotypes and genotypes across the population as a whole, and between wheat varieties and the presence or absence of individual genes.

With this study, we examine the impact of wheat genotype on both the overall soil metagenome and on an important class of rhizosphere‐associated biocontrol bacteria. Our findings that first year wheat genotype profoundly affects not only the overall *Pseudomonas* population, but also the distribution of individual genotypes in the second year wheat rhizosphere, support the opinion that manipulation of plant‐cropping systems, rather than inoculation with individual bacterial isolates, may hold the key to sustainable pathogen biocontrol.

## Results

### Structure of the soil microbial population

Bacterial isolation and soil sampling took place from the Great Harpenden 2 field site at Rothamsted Research (Harpenden, UK) after a 2 year investigation into the effect of different take‐all inoculum building wheat varieties on crop yield. This field experiment examined the effect of 16 different combinations of first‐ and second year wheat cultivar on take‐all disease prevalence and is described at the following website http://www.wgin.org.uk/stakeholders/newsletters.php. We focussed our analysis on four combinations: first year Hereward [high take‐all inoculum build‐up (TAB)] followed by second year Xi19 (low TAB) or Hereward, and first year Cadenza (low TAB) followed by second year Hereward or Xi19 (McMillan *et al*., 2011). At the end of the second year, the plots planted with Cadenza in the first year displayed significantly lower levels of take‐all disease than plots planted with Hereward in the first year (*P* = < 0.001). It was also found that first year Hereward plots had a significantly lower grain yield than first year Cadenza plots (*P* = < 0.001). Consequent to these observations, we observed a strong negative correlation between take‐all infection and grain yield in the second year wheat plots (r = −0.903, *P* = < 0.001).

**Figure 1:**
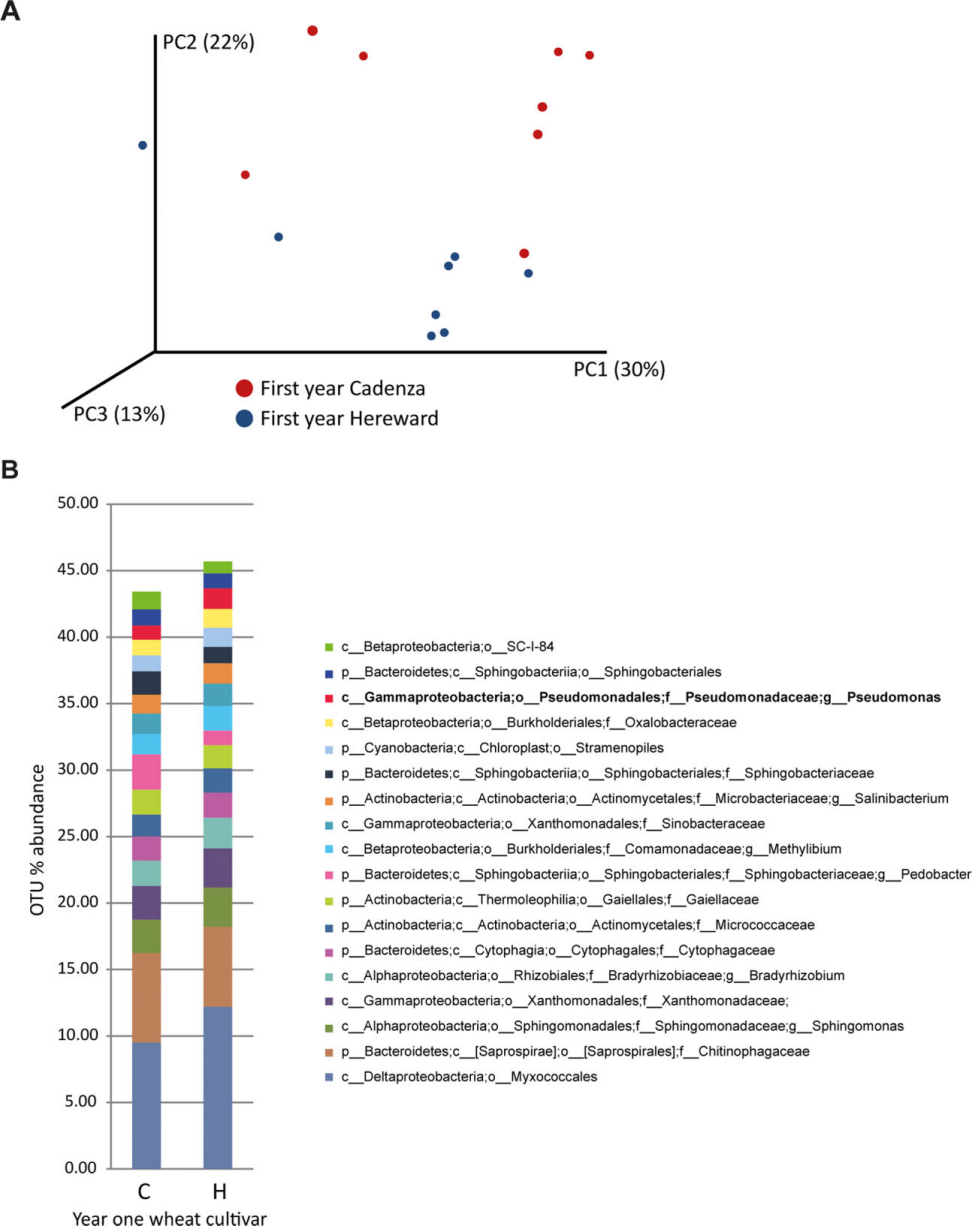
A. Principal component analysis of second wheat rhizosphere communities: Plot is based on amplicon sequencing of a fragment of the 16S rRNA gene sequence. B. Taxonomic analysis of wheat rhizosphere bacteria as shown from 16S rRNA gene amplicon sequencing. The average bacterial abundance is measured for rhizosphere bacterial groups according to the first wheat crop cultivar. OTU% = Percentage of total operational taxonomic units detected.

Next, we assessed the overall rhizosphere soil bacterial population at the end of the second year in these plots using a 16S rRNA gene fragment next‐generation amplicon sequencing approach. Principal component analysis of this data identified an association of community profiles according to first wheat variety (Fig. 1A), which was explained by a cumulative increase in the total population of the seven most abundant operational taxonomic units (OTUs; *Myxococcales*, *Chitinophageacea*, *Sphingomonas*, *Xanthomonas*, *Bradyrhizobium, Cytophagacease* and *Gaiellaceae*) from 26.8% ± 0.4% after first year Cadenza to 30.5% ± 0.6% after first year Hereward. (Fig. 1B). This observation was positively correlated with year 2 take‐all infection index (r = 0.64, *P* = 0.008) and negatively correlated with grain yield (r = 0.55, *P* = 0.029). Furthermore, analysis of the 20 OTUs with an abundance of 1% or more of the total bacterial community revealed that the abundance of 4 OTUs was significantly influenced by first year wheat variety. Of these, a group belonging to the Betaproteobacteria; Order SC‐I‐84 was more abundant in first year Cadenza plots (*P* = 0.025). The other three OTUs were more abundant following first year Hereward: a group in the family *Oxalobacteraceae* (*P* = 0.049), a group in the family *Sphingomonadaceae* (*P* = 0.049) and finally the *Pseudomonas* spp., which were the most significantly influenced group by first year wheat variety (*P* = 0.002), with an average abundance of 1% ± 0.002% after first year Cadenza and 1.69% ± 0.002% for first year Hereward plots (Fig. 1B). This observation, combined with the established links between *Pseudomonas* and biocontrol, led us to investigate this species group in more detail.

### Phenotypic and phylogenetic variation among *P* *. fluorescens* isolated from wheat fields

**Figure 2:**
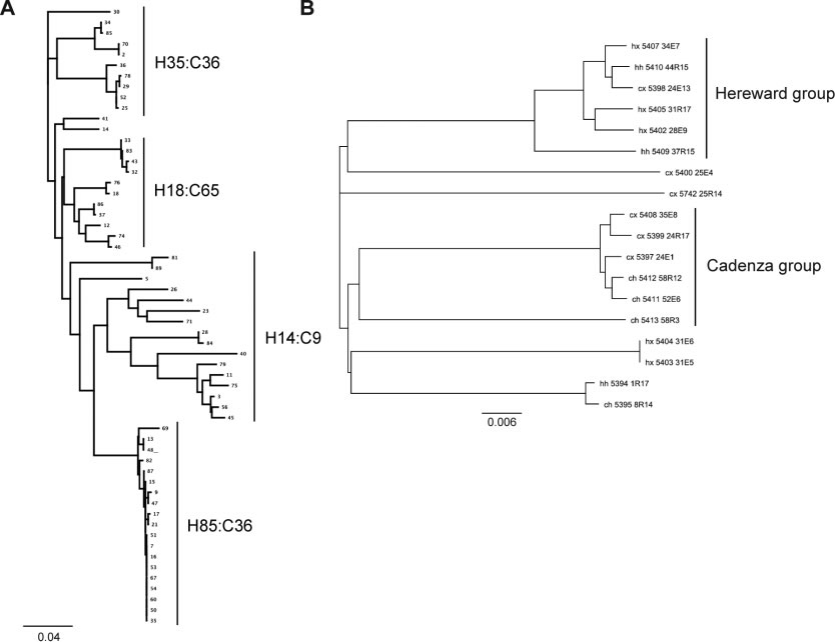
A. Phylogeny of *P* *. fluorescens* genotypes based on *gyr* *B*: A total of 318 isolates were isolated from the field experiment and fingerprinted using ERIC PCR. Groupings were confirmed by sequencing of a fragment of the *gyr* *B* gene. The text associated with the braced groups shows the number of isolates in each group that was derived from first year Hereward (H) or Cadenza (C) plots. B. Phylogenetic tree based on an 8 gene concatenation from 18 genome sequenced *P* *seudomonas fluorescens* isolates. The code for each node reads: wheat cultivar combination in year 1: Hereward (h) or Cadenza(c); followed by year 2: Hereward (h) or Xi19 (x); genome sequence library number; isolate code: plot number followed by rhizosphere (R) or endosphere (E) source of isolation.

A total of 318 *Pseudomonas* isolates were collected from the rhizosphere and root endosphere of wheat plants under the different test conditions. Approximately half of the isolates were obtained from the endosphere and half from the rhizosphere compartment. Approximately 10^5^ *P. fluorescens* colony‐forming units (CFU) were identified in the rhizosphere per gram of soil and 10^4^ CFU in the endosphere per gram of root tissue. No significant differences were found between the rhizosphere and endosphere samples in terms of colony counts (data not shown). Isolates were characterized by ERIC PCR, and groupings confirmed by sequencing fragments of the *gyrB* gene from which a phylogenetic tree was built (Fig. 2A). The phylogenetic analysis was based on *gyrB* as opposed to 16S rRNA sequence to enable finer resolution of different isolates (Yamamoto and Harayama, 1995). The isolates fell into four main groupings (Fig. 2A). When the tree was populated with abundance data, the two most populated groups were found to be dominated by first wheat Cadenza isolates (78% of 121) and Hereward isolates (70% of 83). These data show that in addition to influencing the overall abundance of OTUs within the *Pseudomonas* genus, first year wheat pre‐treatment of the soil exerts selective pressure on *Pseudomonas* associated with the second wheat at the level of individual bacterial genomes.

In order to more closely examine the genetics of the wheat rhizosphere‐associated *Pseudomonas* spp., 19 representative isolates (9/10 randomly selected from year 1 Hereward and year 1 Cadenza plots respectively) were fully genome sequenced. An initial phylogenetic analysis of 18 of these (one genome did not meet the requirements for phylogenetic analysis and was excluded) using a concatenation of 8 single copy housekeeping genes (*aroE*, *atpD*, *dnaE*, *guaA*, *gyrB*, *mutL*, *pyrC* and *recA*) agreed with the much more extensive *gyrB* phylogenetic tree above (Fig. 2A). The majority of genomes clustered into two main groups, each comprising six isolates (Fig. 2B). The first was made up entirely of isolates from first year Cadenza plots, whereas in the second, 5 of 6 isolates were from first year Hereward plots, confirming that these 19 isolates are representative of the larger genotypic data set.

Visual inspection of the 318 *Pseudomonas* strains revealed extensive phenotypic variation, with a range of different colony morphologies and visible‐secreted molecules present across the whole field sample. To effectively assess this phenotypic information, 55 isolates were randomly selected and assayed for several phenotypes relevant to rhizosphere colonization and biocontrol. Relative ordinal values were collected for growth rate (RGR), swarming motility (motility), the secretion of visible (H72) and UV fluorescent (H24) molecules, Congo Red (CR) binding (a generic test for surface attachment factors; Spiers *et al*., 2003) (CRB) and the ability to suppress the growth of take‐all fungus (TAR) as well as two well‐studied model actinomycete species, *Streptomyces coelicolor* (SCR) and *Streptomyces venezualae* (SVR). Much of the *Pseudomonas* accessory genome is devoted to antibacterial pathways (Loper *et al*., 2012), and bacterial antagonism is undoubtedly a major factor in ecological success in the rhizosphere. The actinomycetes are one of the most abundant classes of microorganisms in the soil and rhizosphere and an important natural source of clinical antibiotics (Watve *et al*., 2001), hence their inclusion in this study. Phenotypic data are summarized in Table S1.

**Figure 3:**
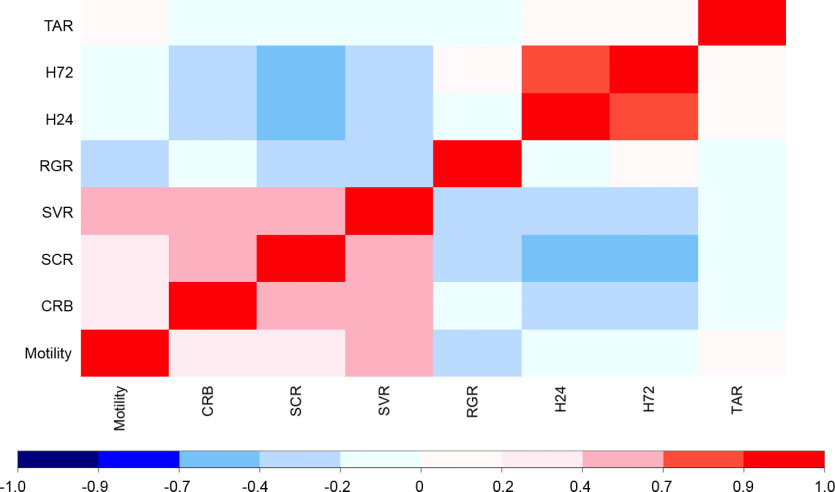
‘Phenotype’ versus ‘phenotype’ correlation coefficient analysis: Spearman's rank correlation coefficients between the 10 measured bacterial phenotypes. Acronyms used are as follows: TAR – take‐all resistance, H72–72 h visible siderophore production, H24–24 h UV siderophore production, RGR – relative growth rate, SVR – *S* *treptomyces venezualae* suppression, SCR – *S* *treptomyces coelicolor* suppression, CRB – Congo Red binding. The scale range is from dark blue (highly negatively correlated phenotypes) to bright red (highly positively correlated). Light colours represent low correlations.

**Figure 4:**
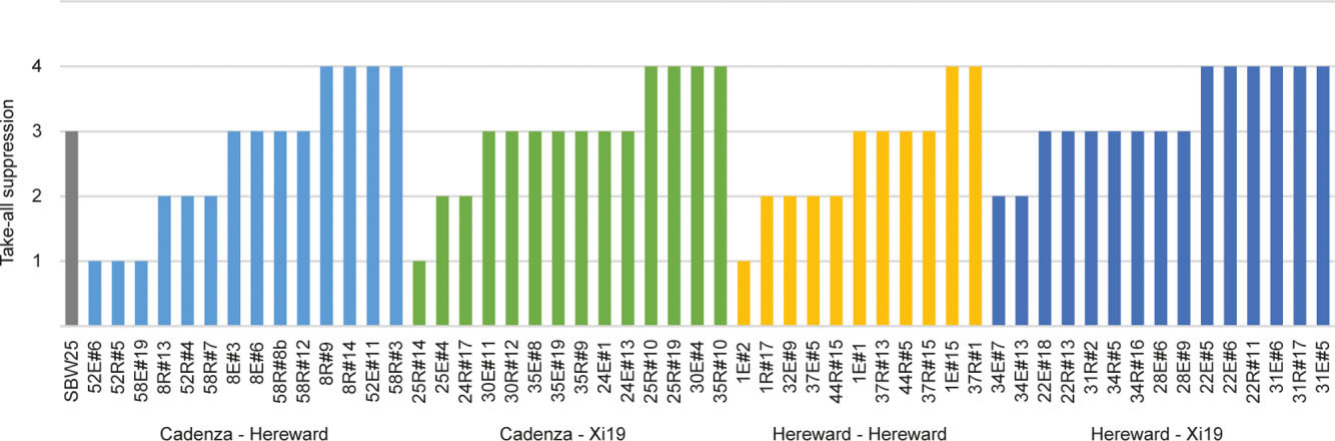
Take‐all suppression by wheat field *P* *seudomonas* isolates: Graph shows the relative extent of take‐all suppression for the 55 *P* *seudomonas fluorescens* isolates in this study. Ordinal values were assigned from 1 (no suppression of fungal growth) to 4 (strong suppression) and are clustered according to field site of isolation. The well‐characterized model organism *P* *. fluorescens* SBW25 was included as a comparator and was assigned a value of 3. The graph shown here is a representative of two independently run experiments.

In order to identify any relationships between different bacterial phenotypes (Table S1), Spearman's rank correlation coefficients were applied to the phenotypic data sets (Fig. 3). In addition to several strong but unsurprising correlations, e.g. for suppression of the two related *Streptomyces* spp., we observed a number of unexpected correlations between individual phenotypes. Several *P. fluorescens* phenotypes correlated, both positively and negatively, with suppression of *Streptomyces* growth and development. A negative correlation was observed between *S. coelicolor* growth suppression (SCR) and the production of UV/visible secreted molecules (H24 and H72). Conversely, increased levels of CRB and swarming motility were positively correlated with actinomycete suppression (SCR and SVR). However, despite both apparently contributing to the ability of *Pseudomonas* to compete against *Streptomyces*, motility and CRB did not significantly correlate with one another. Surprisingly, in spite of substantial observed variation in take‐all suppression across the population, with numerous isolates exerting a strong suppressive effect on take‐all growth (Fig. 4), no significant correlations were seen between take‐all suppression and any of the other tested phenotypes. This suggests that the mechanism(s) employed by *Pseudomonas* to suppress soil take‐all are selected for independently of other phenotypic parameters tested here.

### Correlations between phenotypes and phenotypic output genes

**Figure 5:**
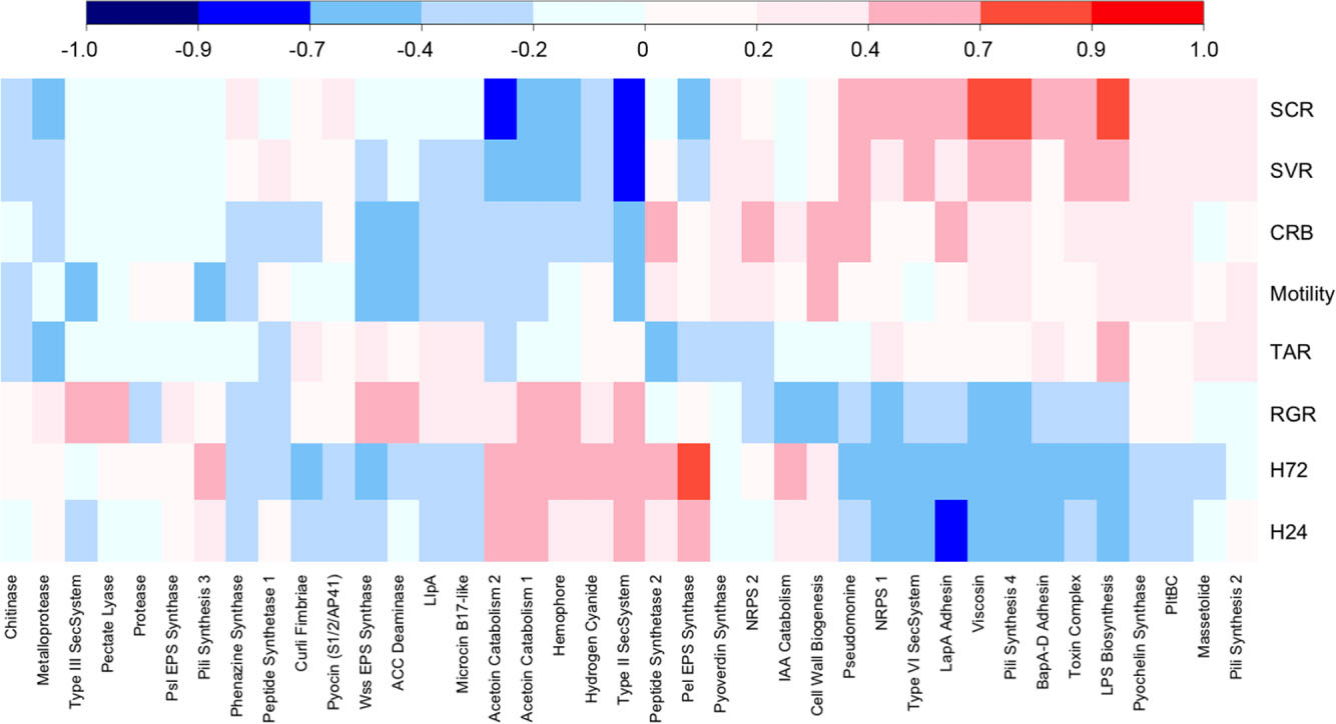
‘Phenotype’ versus ‘phenotypic output gene’ correlation coefficient analysis: Spearman's rank correlation coefficients between the 10 measured bacterial phenotypes and the presence or absence of phenotypic output operons. Acronyms used for phenotypes are the same as described for Fig. 3. The scale range is from bright red (highly positively correlated) to dark blue (highly negatively correlated). Light colours represent low correlations.

Next, we examined the relationship between the measured *Pseudomonas* phenotypes and the presence or absence of well‐characterized phenotypic output loci in the 19 sequenced *P. fluorescens* genomes. A total of 376 individual genes from 61 operons were examined. In 22 cases, little or no variation was observed between the genomes for the presence or absence of the tested operon (Table S2), whereas for the remaining 39 loci Spearman's rank correlation coefficients were applied as before (Fig. 5). Strong positive correlations were observed between *Streptomyces* suppression and the presence of genes for type IV pili and the surfactant viscosin (contributing to swarming motility) and the Wsm lipopolysaccharide (LPS) biosynthesis and LapA/BapA adhesin operons (CRB). Conversely, the presence of another CRB pathway, the *pel* exopolysaccharide (EPS) biosynthesis operon, negatively correlates with *Streptomyces* suppression (Fig. 5). Significant positive correlations were also observed between *Streptomyces* suppression and specific operons linked to cell killing, including loci for toxin/secondary metabolite production and type VI secretion.

A positive correlation was observed between UV/visible siderophore secretion and genes for the haem‐scavenging protein hemophore (Wandersman and Delepelaire, 2004), suggesting that these two iron‐scavenging pathways are co‐selected in the wheat rhizosphere. Furthermore, a number of additional connections were identified where the relationship between the conserved genes and the phenotype in question was less clear. For example, positive correlations were seen between UV/visible siderophore secretion and genes associated with plant‐growth manipulation (auxin and acetoin catabolism), type II secretion, *pel* EPS synthases, and various secondary metabolite biosynthesis operons including hydrogen cyanide production (Fig. 5). *Pseudomonas fluorescens* growth rate showed a positive correlation with several operons required for plant manipulation, cell killing and the degradation of plant material. These include genes for pectate lyase production, type II and type III secretion, ACC deaminase and acetoin catabolism (Fig. 5). Growth rate was also positively correlated with the presence of the *wss* EPS operon (Spiers *et al*., 2003), although the reasons for this are currently unclear. In agreement with the phenotypic analysis above (Fig. 3), few significant correlations were seen between take‐all suppression and the loci included in this study.

### Correlations between phenotypic output genes: the internal structure of *P* *seudomonas* genomes

**Figure 6:**
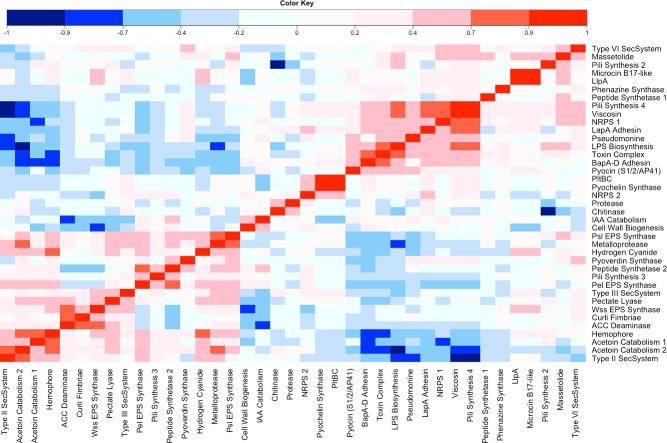
‘Output gene’ versus ‘output gene’ correlation coefficient analysis: Spearman's rank correlation coefficients between the individual phenotypic output operons. The scale range is from dark blue (highly negatively correlated) to bright red (highly positively correlated), Light colours represent low correlations.

To further define the parameters of *P. fluorescens* genome structure, the correlation coefficient analysis was expanded to compare the presence/absence of phenotypic operons with one another (Fig. 6). In agreement with our earlier findings (Fig. 5), we identified strong positive correlations between the operons that are suggested to contribute to *Streptomyces* suppression. The presence of type IV pili genes strongly correlates with the viscosin synthase operon, along with non‐ribosomal peptide synthetase (NRPS), *wsm* LPS synthesis and toxin complex genes. Furthermore, all five loci were strongly negatively correlated with genes for hemophore, acetoin catabolism and type II secretion. This second set of genes also positively associates with one another, with genes for hemophore and hydrogen cyanide synthesis also sharing a strong positive correlation (Fig. 6).

The examination of the correlation analyses as a whole supports the existence of several discrete sub‐groups of phenotypic loci within the *Pseudomonas* population. The first group (containing genes for viscosin, pili, *wsm* LPS etc.) are effective for actinomycete suppression, but produce few siderophores or plant‐growth manipulation enzymes. Conversely, the second group (hemophore, acetoin catabolism etc.) produces and secretes siderophores and other small molecules, but have limited antibacterial capability. Interestingly, this pattern is also seen for the phenotypic correlation analysis in Fig. 3. In this experiment, actinomycete suppression and siderophore production negatively associate with one another across a large number of isolates, suggesting that these broad phenotypic groupings are also present in the wider *Pseudomonas* population.

Less‐easily identified in the phenotypic data, a potential third strongly associating group comprises a subset of those genes associated with enhanced growth rates (Fig. 5). The ACC deaminase gene was strongly positively correlated with both the *wss* cellulose synthase and curli fimbriae operons, and to a lesser extent with genes for pectate lyase (*pel*) synthesis. Interestingly, a strong negative correlation was also seen between ACC deaminase and another plant manipulation locus: IAA (auxin) catabolism. Consistent with the positive correlation data, this negative association is mirrored for the *wss*, curli and *pel* operons, alongside type III secretion system genes (Fig. 6).

### The effect of environmental parameters on *P* *seudomonas* gene ontology in the wheat root environment

**Figure 7:**
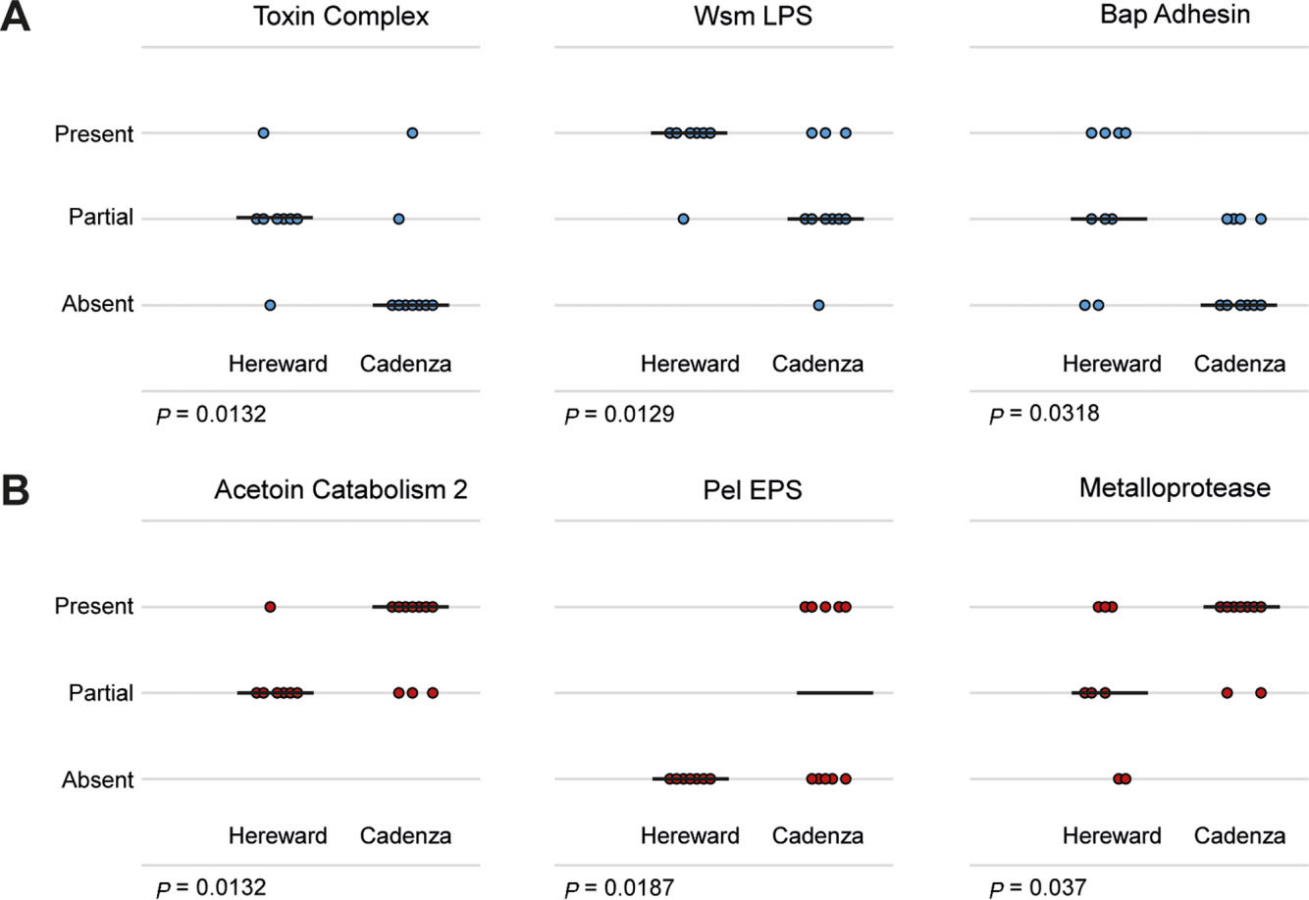
Impact of first year wheat variety on the abundance of *P* *seudomonas* phenotypic output loci: Wilcoxon–Mann–Whitney tests to probe the genetic differences between strains isolated from the wheat varieties cultivated in year 1. Each dot represents 1 of the 19 *P* *. fluorescens* sequenced genomes interrogated for the presence or absence the phenotypic output operons noted in each case and is reported as present, partially present or absent. Operons that were significantly more abundant in first year Hereward plots are shown in blue (A), those that were more abundant with year 1 Cadenza in red (B). The two‐tailed *P* value from the Mann–Whitney test is reported beneath each box. For visibility purposes only, some noise was introduced to the positions of the points to avoid their superposition.

The genotyping analysis in Fig. 2A shows that the selective pressure exerted on the soil microbiome by different first year wheat varieties impacts not only at the level of microbial population structure, but also on the distribution of individual *P. fluorescens* genomes. This first year selective pressure can also be seen in the phylogenetic trees of sequenced *Pseudomonas* isolates (Fig. 2B), prompting us to look for evidence for selective pressure from year 1 wheat variety on the phenotypic output loci present in these genomes. For the 39 variable phenotypic loci (see above), Mann–Whitney tests were applied to probe the impact of wheat variety cultivated in year 1 or year 2 on the presence of each operon. Multiple significant connections were seen between the wheat variety planted in year 1 and the presence or absence of phenotypic output operons. The presence of the *wsm* LPS biosynthesis operon, genes for toxin production and the *bapA* adhesin correlated strongly (*P* < 0.05) with year 1 Hereward cultivation (Fig. 7A). Conversely, metalloprotease and acetoin catabolism genes, and the *pel* exopolysaccharide operon were significantly more abundant in strains isolated from year 1 Cadenza plots (Fig. 7B). By contrast, only two loci correlated significantly with the wheat varieties cultivated in year 2. Genes for indole acetic acid (auxin) catabolism were significantly more abundant in strains isolated from year 2‐Xi19 plots (*P* = 0.0194), whereas isolation from Hereward plots in year 2 positively correlated with the pectate lyase gene (*P* = 0.011). The difference in selective pressure between year 1 and year 2 mirrors the results seen for the wider phylogenetic analysis of soil isolates (Fig. 2A), with six operons significantly associating with year 1 wheat genotypes as opposed to only two with year 2.

The three operons associated with the cultivation of Hereward in year 1 all positively correlated with each other and with actinomycete suppression (Figs 5 and 6). Conversely, those loci whose presence positively correlated with year 1 Cadenza strongly associated both with each other and with increased siderophore production (Figs 5 and 6). A positive association was also seen between year 1 Cadenza and siderophore production in the 55 isolate phenotypic data set, although this connection was not statistically significant (*P* = 0.13). Given the small size of our genomic data set, it is important not to overstate conclusions at this stage about the relationship between particular *Pseudomonas* phenotypic loci and either wheat genotype or take‐all suppression. Nonetheless, these analyses identify several novel, potential gene targets for interrogation across the wider *Pseudomonas* rhizosphere population, and suggest clear directions for future experimental work.

## Discussion

Most studies to date of *Pseudomonas* genomic and phenotypic diversity in soil have been conducted using model organisms isolated at different times and from different plant/soil environments (Silby *et al*., 2009; Loper *et al*., 2012), as part of wider metagenomic analyses of whole soil microbiomes (Mendes *et al*., 2011; Bulgarelli *et al*., 2012), or with a focus on individual loci, for example 2,4‐DAPG (de Souza *et al*., 2003). More recently, Mavrodi and colleagues investigated the geographic distribution of phenazine‐producing *Pseudomonas* species in US wheat fields (Mavrodi *et al*., 2012; Parejko *et al*., 2012), applying a population genetics approach to show that low precipitation selects for Phz + Pseudomonads (Mavrodi *et al*., 2012) and demonstrating the utility of such methods for the analysis of soil microbe populations. In this study we integrate elements of these different approaches to examine how a defined environmental change influences rhizosphere microbial diversity. More specifically, we use a combination of microbiological assays, next‐generation sequencing and statistical analysis to examine how the presence of wheat varieties with different take‐all inoculum building characteristics (McMillan *et al*., 2011) impacts on the distribution of phenotypes and genotypes in the important soil bacterium *P. fluorescens*.

The *P. fluorescens* species group (including *P. protegens*, *P. chlororaphis* and other sub‐species) displays very extensive genomic diversity, with a core genome of around 2800 genes supplemented by an accessory genome of 2500–3500 genes (Silby *et al*., 2009; Loper *et al*., 2012; Redondo‐Nieto *et al*., 2012; Seaton and Silby, 2014). This genomic diversity manifests in a similarly extensive range of different phenotypes (Loper *et al*., 2012), and in turn into marked differences in plant colonization efficiency and biocontrol ability (Naseby *et al*., 2001). In agreement with previous soil/rhizosphere metagenomic studies, amplicon sequencing of the Great Harpenden 2 field reveals a highly complex and diverse microbial community (Mendes *et al*., 2011; Bulgarelli *et al*., 2012). Subsequent genomic analysis indicated that this diversity extends not only to the level of species/genus, but also to variability within the *P. fluorescens* species group, with genetic diversity in our single experimental field site only slightly lower than that recorded for all *P. fluorescens* strains annotated to date (Loper *et al*., 2012; Seaton and Silby, 2014).

Genetic variation was observed both at the single nucleotide level for core housekeeping genes, and in terms of the variability present in the accessory genome. The pangenome for the 19 sequenced strains in our experiment contains 9907 predicted protein‐coding open reading frames, compared with previously published values of 13 872 (Loper *et al*., 2012) and 13 914 (Seaton and Silby, 2014) for all annotated *Pseudomonas* strains. This pangenome encodes an extensive array of phenotypic outputs and secondary metabolite operons, including a substantial proportion of the *Pseudomonas* output pathways characterized to date. In total, 82% of the phenotypic output operons included in our analysis appeared at least once in the 19 genome sequences from our field site. As this screen was based only on characterized loci found in the published literature, these genomes undoubtedly contain a significant number of additional, as‐yet uncharacterized output pathways.

16S rRNA gene amplicon sequencing of the four wheat field conditions identified substantial selective pressure on the soil metagenome arising from differences between the two wheat varieties cultivated in the first year. First year Hereward wheat plots have higher take‐all inoculum build‐up and display an increased abundance of saprophytes in second year wheat plants compared with first year Cadenza. This is presumably due to the premature senescence of diseased roots in these plots, resulting in increased nutrient release in the root zone, which saprophytes are well adapted to assimilate. Excitingly, the most substantial difference in the amplicon data between year 1 Hereward and Cadenza samples was seen for the *Pseudomonas* genus, with significantly more *Pseudomonas* OTUs identified with Hereward in year 1.

Following on from the 16S amplicon experiment, we used a combination of extensive genotyping analysis, alongside the statistical examination of smaller genomic and phenotypic data sets to examine the effect of planting different wheat varieties on the rhizosphere *Pseudomonas* population in more detail. Once again, the wheat variety planted in year 1 exerted significant selective pressure on the rhizosphere *P. fluorescens* genome. Evidence of year 1 wheat selection was seen with the ERIC profiling and *gyrB* sequence data (Fig. 2A), as well as in the phylogenetic analysis of housekeeping genes (Fig. 2B), and the presence or absence of accessory genome elements (Fig. 7). Compared with the selective pressure exerted by the first year wheat, the second year wheat cultivars had comparatively little effect on the *Pseudomonas* genomes in our study.

Our data suggest that environmental selection can exert marked effects on the relative abundance of different *Pseudomonas* genotypes in the rhizosphere environment. The presence of selective pressure from year 1 wheat changes not only the overall abundance of *Pseudomonas* spp. in the soil, but also impacts on *Pseudomonas* metagenome structure, with individual genotypes becoming more or less abundant within the overall population. In complex ecosystems like soil, adaptive responses at this level would be missed by conventional amplicon or metagenomic analysis. While the genomic data set examined here is relatively small, the results of the phylogenetic and statistical analyses of these samples are in full agreement with the far more extensive ERIC PCR and *gyrB* phylogenies (Fig. 2A), and the correlation analyses of phenotypic data (Fig. 3) described elsewhere. This increases our confidence that the evidence for structure and selective pressure we see in the genomes of our sequenced isolates is indeed representative of the larger sample sets.

In addition to mapping the impact of selective pressure on the microbial genome, our analysis sheds light on the genome structure of soil *Pseudomonas* spp. and the relationship between phenotypes and genotypes in the rhizosphere environment. Despite the vast potential within the pangenome to produce novel and diverse phenotypic combinations, the wheat *Pseudomonas* genomes showed a significant degree of internal structure, with discrete clusters of phenotypic genes emerging, for example the LPS, pili synthesis and viscosin operons. These clusters were generally associated with a particular broad behavioural characteristic, for example microbial suppression, plant association/manipulation or scavenging and growing on plant material. Operons within these phenotypic clusters were strongly co‐selected with each other, despite often being located in distant regions of the genome. As well as the positive correlations seen between sets of phenotypic genes, strong negative correlations were observed between the loci in one phenotypic cluster and those in another.

The microbial community selection observed in these experiments is clearly being driven by the plant genotype in the first year. While as yet we do not understand how this is linked to take‐all infection in the second year crop, it is interesting to speculate as to how the plant recruits and shapes the root microbiome from the bulk soil microbial reservoir, and whether these microbes are subsequently important for disease control. It is possible that the low TAB cultivars directly suppress fungal pathogen load through the root exudation of antifungal compounds and that the bacterial and *Pseudomonas* community structures observed in these experiments are simply bioindicators of this. However, the putative connections observed between first year Hereward plants and actinomycete suppression, and first year Cadenza plants and enhanced siderophore production and titre of plant association genes suggest that the system is complex and multi‐trophic. The results imply that following the growth of the high TAB Hereward variety in year 1 there is an increase in the abundance of *P. fluorescens*, which are better adapted to fighting other microbes, in order to effectively colonize senescent root tissue, whereas after growth of the low TAB variety Cadenza in year 1 we observe a shift towards genotypes that are better adapted to communicate with the plant host and are also able to produce a greater amount of siderophores, which could be important in preventing fungal pathogen establishment (Kloepper *et al*., 1980). Manipulative experiments in gnotobiotic conditions are required to examine how cultivar‐specific root chemistry influences the system.

This research demonstrates the importance of analysing the genetic and phenotypic variation inherent within individual species, as well as that found at the species/genus level, when studying complex microbial systems such as plant rhizospheres. The approach we have taken, enabled by high‐throughput next‐generation sequencing, allows us to analyse selection and variability in the microbiome at a far higher resolution than has previously been attempted.

## Experimental procedures

### Field trial and sampling

The field trial was located at the Rothamsted Research experimental farm, UK (N51°48′27″, W0°21′44″), and took place for 2 years from 2011 to 2012 (field trial 11‐12/R/CS/719). In the autumn of 2010 plots were either sown with high (Hereward) or low (Cadenza) take‐all inoculum build‐up cultivars. In autumn 2011, plots were over‐sown with either Hereward or another commercial winter wheat cultivar, Xi19. The second year plots were derived from the following cultivar combinations: Hereward/Hereward; Hereward/Xi19; Cadenza/Hereward; Cadenza/Xi19. Seeding was at 350 seeds/m^2^ on each of 16 10 m × 3 m plots (4 replicates × 4 soil culture treatments). The take‐all intensity index (TAI) (Bateman *et al*., 2004) was used to characterize the severity of take‐all disease (‘fungal pathogen load’) in year 2. From each plot, five wheat plants at late milk growth stage were sampled in a ‘w’ formation across the plot to a depth of around 30 cm with crown roots and a proportion of the seminal roots attached. Plants were placed in plastic bags and transported back to the laboratory. The field trial was harvested and fresh grain weights for mature plants recorded in August 2012. Grain dry matter was determined by oven‐drying 80 g of sub‐samples of the fresh grain. Grain yields were adjusted to 85% dry matter and extrapolated to tonnes per hectares.

### Rhizosphere harvesting

Bulk soil was shaken from each plant and the root systems were cut into 2–3 cm sections and mixed by shaking. A 10 g subsample was transferred to a 50 ml Falcon tube and 30 ml sterile water added. The roots were vortexed vigorously for 90 s to release the rhizosphere soil from the root system. Tubes containing rhizosphere soil suspensions were centrifuged at 4000 r.p.m. for 10 min at 4°C, and the supernatant discarded. The root systems were transferred to fresh tubes and endosphere isolates were recovered according to the method described by Robinson and colleagues (2015). Serial dilutions of both rhizosphere soil and endosphere samples were plated onto *Pseudomonas* CFC selective agar (Oxoid) and incubated at 27°C until colonies emerged.

### Phylogenetic analysis I – ERIC and *gyrB* sequence profiling

Up to 15 colonies from both the rhizosphere and endosphere of each sample were randomly sampled to represent the plant‐associated *Pseudomonas* diversity of each plot. Isolates were lysed and genomic DNA released with MicroLysis + Microzone (UK), as described by the manufacturer's instructions. Bacterial genotypes were determined for each isolate by ERIC PCR with primers as described by Versalovic and colleagues (1991). ERIC profile groupings were established and a member of each type used as the template for *gyrB* PCR with primers described by Yamamoto and colleagues (2000). The amplified *gyrB* PCR products were confirmed by electrophoresis, and sequenced using the forward and reverse primers described by Yamamoto and Harayama (1995). Sequences were tested with the blastn algorithm using default settings to ensure their identity as *P. fluorescens*. PCR product DNA sequence pairs for the *gyrB* gene fragments were then used to create ‘*in silico*’ molecules in Geneious version 6.1.6. The *gyrB* sequences are deposited in the GenBank Nucleotide database with accession numbers KF059880‐KF059937. For phylogenetic analysis a 940 bp fragment for each distinct genotype in the study was used to construct a phylogeny using Simultaneous Alignment and Tree Estimation (Liu *et al*., 2012).

### Phenotypic assays

Unless otherwise stated, all *P. fluorescens* strains were grown at 28°C in Lysogenic Broth (LB) medium (Miller, 1972), and KB medium (King *et al*., 1954) solidified with 1.3% agar where appropriate. The CRB assay was adapted from Spiers and colleagues (2003). Five 10 μl drops of LB overnight cultures per strain were grown on 20 ml KB agar plates for 24 h at 28°C. Each colony was then re‐suspended in 1 ml 0.005% (w/v) CR (Sigma) and incubated for 2 h at 37°C with shaking. Colony material was pelleted by centrifugation and CR remaining in the supernatant determined by measurement of A_490_ compared with appropriate CR standards. CRB was expressed as a fraction of the CR bound by SBW25. To measure swarming motility (Motility), 0.3% KB agar plates containing 0.1% NaCl and 0.004% CR dye were poured and allowed to set and dry for 1 h in a sterile flow chamber. Plates were then inoculated with 2 μl of spots of *P. fluorescens* overnight cultures, and incubated overnight at room temperature. Each sample was tested in triplicate. Growth curves (RGR) were measured in 96‐well plates containing 150 μl LB/well and inoculated with LB overnight cultures to an initial OD_600_ of 0.1. Optical density was measured every hour for 24 h at 28°C without shaking. To measure UV/visible siderophore production, 10 μl of LB overnight cultures were spotted onto 25 ml M9 0.4% pyruvate agar plates and incubated for 72 h at 28°C. Visible siderophores (H72) were assessed after 72 h, whereas UV fluorescence (H24) was measured from photographs taken after 24 h with a UV filter. *Streptomyces* cross‐streak assays were carried out on MYM (SVR) or SF + M (SCR) agar (Kieser and John Innes Foundation, 2000) as appropriate. Two parallel lines of *Streptomyces* spores were streaked onto each plate. These lines were then cross‐streaked with *Pseudomonas* isolates grown overnight in LB. Plates were incubated at 30°C and the relative performance of each species assessed daily for 72 h. Take‐all inhibition assays (TAR) were conducted with *G. graminis var. tritici* strain NZ.66.12 (isolated from Rothamsted New Zealand field in 2012). Three 10 μl drops of LB overnight cultures per strain were placed equidistantly 15 mm from the edge of Potato Dextrose Agar (PDA) plates and incubated for 24 h at 28°C. A 3 mm plug from the leading edge of an NZ.66.12 culture was then placed in the centre of each plate and incubated for a further 5 days at 22°C before the extent of *Ggt* inhibition by *P. fluorescens* was assessed.

### Amplicon sequencing

For each rhizosphere soil sample, DNA was isolated from 0.25 g of soil using the Mo Bio PowerSoil™ DNA Isolation Kit (Carlsbad, CA, USA). Extractions were performed according to the manufacturer, but with the use of an MP Biomedicals FastPrep‐24 machine for 30 s at 5.5 m/s. Genomic DNA concentration and purity were determined by NanoDrop spectrophotometry (Thermo Scientific, Wilmington, DE, USA). Bacterial and archaeal 16S rRNA genes were amplified from rhizosphere soil DNA samples with barcoded universal prokaryotic primers (515F/R806) targeting the V4 region, subjected to Illumina® sequencing using the MiSeq platform to generate 2 × 150 bp paired‐end reads, and analysed using the qiime 1.8 pipeline (utilizing greengenes for taxa classification and the uclust algorithm for OTU clustering), as described by Caporaso and colleagues (2010; 2011). Specifically, paired‐end reads were merged prior to analysis and no mismatches were allowed in barcode and primer sequences, a Phred score of 30 or above was adopted, chimeras were removed and one ambiguous base was allowed in the remaining trimmed sequence. A total of 38 175 reads were sub‐sampled from each of 16 samples (a total of 610 800 reads). Reads were assigned to bacterial genera (where possible) with 97% sequence identity.

### Illumina® genome sequencing

A total of 19 strains representative of the breadth of the *gyrB* tree were selected for whole genome sequencing. Genomic DNA was extracted from target strains grown overnight in LB using a GenElute™ Bacterial Genomic DNA Kit (Sigma), quantified by nanodrop spectroscopy and submitted to Genome Enterprises Ltd. (TGAC) for Illumina barcoded TruSeq library construction and sequencing on one lane of an Illumina® HiSeq 2500 platform (Rapid‐Run mode) using 150 bp paired‐end reads. Genome sequences were then automatically assembled by TGAC and subjected to bioinformatic analysis. Assemblies have been submitted to the European Nucleotide Archive (http://www.ebi.ac.uk/ena/data/view/<accession‐numbers>) with the accession numbers given in Table S3.

### Bioinformatic analysis

Based on the sequence assembly and annotation provided by TGAC, protein sequences were derived and stored in separate fasta files for each of the 19 isolate genomes, and 4 reference genomes downloaded from GenBank (Pf‐5, Pf01, F113 and SBW25). Genes encoding proteins of interest were selected from within the reference genomes by a combination of blast similarity searching and searches for Pfam domains of interest in the translated proteomes. All Pfam searches were carried out against Pfam‐conserved domains profiles downloaded from the Conserved Domains Database (CDD) on the NCBI website using the rpsblast program in the NCBI Blast+ suite (version 2.2.28). Proteins of interest in each of the reference genomes were then blast‐searched against all the other genomes individually, and reciprocal blast hits were stored in a relational database. Finally, genomes were manually examined for the presence, partial presence (defined as between 1% and 50% of loci missing) or absence of individual genes and operons. Decisions were made based on a combination of sequence identity, alignment coverage, whether or not the hits were reciprocal best hits, and overall operon structure.

### Phylogenetic analysis II – housekeeping genes

Eight single copy housekeeping genes from *P. fluorescens* SBW25 were used as blastn query sequences against the contigs from the 18 sequenced strains. The genes selected were: *aroE*, *atpD*, *dnaE*, *guaA*, *gyrB*, *mutL*, *pyrC* and *recA.* Gene sequences for each isolate were concatenated and phylogenies constructed using the ‘Geneious tree builder’ with default settings in Geneious version 6.1.6.

### Statistical analysis

Analysis of variance was applied to the take‐all infection index, grain yield and bacterial community abundance data to test (using F‐tests) the main effects and interaction between the factors of the first and second year wheat varieties grown. The analysis took account of the split‐plot nature of the designed experiment, the main plots in the three blocks for the first year being split for the varieties grown in the second year. Appropriate means were then compared using the standard error of the difference value on corresponding degrees of freedom, thus invoking the least significant difference at the 5% level of significance. No transformation of the data was required, as residual plots showed that the data conformed to the assumptions of the analysis. The Pearson correlations (*r*) between take‐all infection index, grain yield and bacterial community abundance were also calculated and tested (F‐tests). The GenStat (17th edition, VSN International Ltd, Hemel Hempstead, UK) statistical package was used for these analyses.

For the eight tested phenotypes, each strain was given an ordinal value between 1 (low CRB, slow growth etc.) and 4 (high CRB etc.). For each phenotypic output operon, the sequenced genomes were assigned ordinal values of 0 (locus entirely absent), 1 (partial presence) or 2 (locus present). Ordinal values of 1 (gene present) or 0 (gene absent) were assigned for the tested signalling genes. Loci where no variation was observed between the 19 sample genomes were removed from the study prior to conducting the correlation analysis. All statistical analyses were conducted in r. Correlation analyses were computed using the Spearman's rank method. Krustal–Wallis one‐way ANOVA was used to examine differences between groups. The non‐parametric Wilcoxon–Mann–Whitney test was used to probe the phenotypic and genetic differences between endosphere and rhizosphere isolates, and between isolates from wheat varieties cultivated in year 1 or year 2. All tests were two sided and performed under the null hypothesis that there is no difference between the tested groups. For display purposes, only those signalling genes where more than 10% of the tested genomes varied from the norm were included in the presented figures.

## Supporting information


**Tables S1.** Ordinal values for the colonisation‐associated phenotypes examined in this study.
**Tables S2.** Ordinal values for the presence or absence of genetic loci in the 19 tested *P. fluorescens* genomes.
**Tables S3.** ENA accession numbers for sequenced stains.
